# Supplementary material for: Chimeric systems composed of swapped Tra subunits between distantly-related F plasmids reveal striking plasticity among type IV secretion machines
Source: bioRxiv. 2023 Dec 6:2023.12.05.570194. Preprint. [Version 1] doi: 10.1101/2023.12.05.570194 (PMC10723329; doi:10.1101/2023.12.05.570194)
Supplement: 1 [file NIHPP2023.12.05.570194V1-supplement-1.pdf]

bioRxiv preprint doi: https://doi.org/10.1101/2023.12.05.570194; this version posted December 6, 2023. The copyright holder for this preprint (which was not certified by peer review) is the author/funder, who has granted bioRxiv a license to display the preprint in perpetuity. It is made available under aCC-BY 4.0 International license.

Supporting Information

S1 Fig. Alignments of Tra/Trb protein homologs. Panel A: Alignment of the F and pED208 *tra/trb* regions. **Blue:** VirB/VirD4-like scaffold subunits that are conserved among T4SSs. **Red:** F-specific proteins that contribute to assembly or function of F-like T4SSs. **Yellow:** F-specific proteins involved in surface exclusion. **Green:** Nonconserved proteins. Lines connect genes encoding protein homologs; numbers correspond to the percent amino acid identities across the lengths of the homologs. Accession numbers: pED208 (NCBI Bioproject PRJNA871772); F (NZ_MF370216).

Panels B - F (following pages): Similarities among the Tra/Trb homologs at the primary sequence and structural levels, grouped as indicated (orange highlights). Upper: Sequence alignments of the Tra/Trb proteins indicated, generated with Multalin [79]. Known domains and percent identities are shown for regions indicated. Red lines denote regions of sequence divergence, one or more of which might confer the distinct phenotypes accompanying

bioRxiv preprint doi: https://doi.org/10.1101/2023.12.05.570194; this version posted December 6, 2023. The copyright holder for this preprint (which was not certified by peer review) is the author/funder, who has granted bioRxiv a license to display the preprint in perpetuity. It is made available under aCC-BY 4.0 International license.

subunit swapping described in the text. Lower: Structures of the Tra/Trb homologs predicted from the AlphaFold Protein Structure Database [75]. The solved TraA and $TraB_{ED}$ structures were from RCSB PDB. The structure of pKM101-encoded TraJ was predicted by ColabFold [76]. Structures of the homologs are presented separately and superimposed. For TraB, only the β-barrel and AP domains comprising the OMCC and AP channel are shown. Percent identities of the structurally aligned sequences, Root Mean Square Deviation (RMSD), TM-score, and number of equivalent residues relative to the entire sequence lengths were derived from the RCSB PDB Pairwise Structure Alignment website (https://www.rcsb.org/alignment).

S2 Fig. Functionality of p*oriT* plasmids and *traD* variants. **A.** Transfer of p$oriT_{ED}$ and p$oriT_{F}$ plasmids by donor cells carrying pED208 (blue bars) or F (green bars). **B.** Functionality of Strep-TraD in donor cells with pED208 (blue bars) or F (green bars) variants. Donor cells carried pED208 or F or the isogenic Δ*traD* mutant plasmids without or with plasmids expressing the corresponding *traD* or *strep-traD* genes. **Panels A & B:** Transfer frequencies are presented as transconjugants per donor (Tcs/D). All matings were repeated at least three times in triplicate; a representative experiment is shown with replicate data points and the average transfer frequencies as horizontal bars along with standard deviations as error bars. **Panels C & D:** Host cells carrying pED208Δ*traD* were assayed for production of the strep-tagged $TraD_{ED}$, $TraD_{F}$ or $TraJ_{KM}$ variants shown. Total cellular proteins normalized on a per cell equivalent basis were subjected to SDS-PAGE and immunostaining of western blots with α-strep antibodies for detection of the T4CP variants or α-RNP antibodies against *E. coli* RNA polymerase β-subunit as a loading control.

S3 Fig. Quantitation of strain sensitivities to M13K07. M13K07 phage sensitivity is shown for host cells carrying F or pED208 or gene deletion variants without or with a plasmid expressing the corresponding genes from F or pED208. Phage sensitivity is reported as the number of kanamycin-resistant (Kan^r) transductants per total colony-forming units (CFUs). Panels: **A.** *traD* T4CPs; **B.** IMC subunits; **C.** OMCC subunits; **D.** F-specific components; **E.** *traA* pilins. All infection assays were repeated at least three times in triplicate; a representative experiment is shown with replicate data points and the average transfer frequencies as vertical bars along with standard deviations as error bars. Data in the manuscript figures are presented as '+' (sensitive, defined as $>10^{-4}$

bioRxiv preprint doi: https://doi.org/10.1101/2023.12.05.570194; this version posted December 6, 2023. The copyright holder for this preprint (which was not certified by peer review) is the author/funder, who has granted bioRxiv a license to display the preprint in perpetuity. It is made available under aCC-BY 4.0 International license.

Kan^r colonies/total CFUs), '-' (resistant, defined as <10^{-6} Kan^r/total CFUs), or '+^{p}' (partially sensitive, 10^{-4-6} Kan^r/total CFUs).

S4 Fig. Production of TraB variants and TraA$_{ED}$ pilin in different mutant backgrounds. A. Schematics of TraB chimeras and deletion mutants. Chimeras consist of domains from TraB$_{ED}$ (blue) and TraB$_F$ (green). Host cells carrying pED208Δ*traB* and complementing plasmids were assayed for production of the strep-tagged TraB variants shown. Total cellular proteins normalized on a per cell equivalent basis were subjected to SDS-PAGE and immunostaining of western blots with α-strep antibodies for detection of the TraB variants or α-RNP antibodies against *E. coli* RNP β-subunit as a loading control. **B.** Production of TraA$_{ED}$ pilin in strains carrying pED208 or mutant plasmids deleted of the *tra/trb* genes shown; Lane 2: *E. coli* MC4100 expressing *traA$_{ED}$* from pKKF004 in the absence of pED208. TraA$_{ED}$ pilin was detected by immunostaining with α-TraA$_{ED}$ polyclonal antibodies and the RNP β-subunit with α-RNP antibodies. **C.** Schematic of the pED208 *tra/trb* region with the ΔOMCC and ΔF-specific deletion mutations highlighted. **D.** Production of TraA$_{ED}$ pilin in strains with the pED208ΔOMCC and pED208ΔF-specific mutant plasmids. TraA$_{ED}$ pilin and RNP β-subunit were detected as described above.

S1 Table. Strains and plasmids used in this study.

S2 Table. Oligonucleotides used in this study.
